# Microbial Single-Cell Analysis: What Can We Learn From Mammalian?

**DOI:** 10.3389/fcell.2021.829990

**Published:** 2022-01-17

**Authors:** Zixi Chen, Beixin Mo, Anping Lei, Jiangxin Wang

**Affiliations:** ^1^ Shenzhen Key Laboratory of Marine Bioresource and Eco-Environmental Science, Shenzhen Engineering Laboratory for Marine Algal Biotechnology, Guangdong Provincial Key Laboratory for Plant Epigenetics, College of Life Sciences and Oceanography, Shenzhen University, Shenzhen, China; ^2^ Key Laboratory of Optoelectronic Devices and Systems of Ministry of Education and Guangdong Province, College of Physics and Optoelectronic Engineering, Shenzhen University, Shenzhen, China

**Keywords:** microbes, single-cell analysis, Raman spectra, single-cell sequencing, microfluidics, multi-omics

## Introduction

Traditional microbiology usually studies microbes at population-level, as it is generally assumed that the bulk-cell level assays could describe the status of individuals in the population. However, with recent advances of single-cell level methods, scientists found that heterogeneity of individuals is widely existed among populations (Lidstrom and Meldrum, 2003). Studying the widely existed heterogeneity could help us understand how individuals respond to the environment changes and determine the fate of the whole population (Davis and Isberg, 2016; Roberfroid et al., 2016). In addition, microbiologists could use single-cell level methods to uncover the hidden information in microbes without requiring axenic cultivation, which could greatly expand the scope of target microbes (Stepanauskas, 2012). Here, since several articles have successfully reviewed current methods being applied in microbes (Chen et al., 2017b; Kaster and Sobol, 2020; Liu et al., 2021), we briefly highlighted recent progress on methods for single-cell analysis, including phenotypic and genotypic analysis, especially on those methods which only proved their performance in mammalian cells but showed potentials for microbial applications, and presented perspectives on the future trends of technology development in the field of analyzing microbes at single-cell level.

## Phenotypic Analysis: Non-Invasive Detecting With Spectrum

Rather than randomly sampling, scientists who are interested in single-cell analysis usually prefer to choose individual microbes with specific phenotypes in a complex population for further analysis, if possible, in a high-throughput and non-invasive way. For this purpose, flow cytometry is the most widely used technique, which can detect cells at single-cell level through fluorescence, and help microbiologists distinguish microbes through size, intracellular complexity, DNA/RNA contents, lipid contents, pigment contents, ion concentrations, etc. (Chen et al., 2017b). However, the detection of metabolites and proteins using flow cytometry usually relies on specific stains or antibodies, and may influence the viability of target cells for further axenic culture. To overcome this limitation, several techniques based on Raman spectra have been developed and successfully adapted to microbial single-cell metabolic profiling and phenotypic screening (He et al., 2019; Su et al., 2020; Wang et al., 2020; Xu et al., 2020; Heidari Baladehi et al., 2021; Jing et al., 2021). The profiling of metabolites based on Raman spectrum dispenses with cell fixing or staining, and provides better experimental flexibility and higher cell viability for downstream analysis. In addition, the phenotypic analysis step of both flow cytometry and Raman spectra techniques are high-throughput and can combined with cell sorting system or microfluidics, making it easier for further axenic culture or genotypic analysis (He et al., 2019; Xu et al., 2020; Jing et al., 2021).

## Genotypic Analysis: Single-Cell DNA and RNA Sequencing

Generally, genotypic analysis in microbes includes analyzing DNA and RNA among individuals by revealing the composition and quantification of sequences. For the performance of each methods, our previous work and other reports have summarized the state-of-the-art sequencing methods for microbial single-cell analysis in detail, interested readers could refer to these articles (Chen et al., 2017b; Kaster and Sobol, 2020; Liu et al., 2021). To our knowledge, the main obstacle in this field is to amplify the total DNA from one microbe, but it has been basically solved in the past decade (Chen et al., 2017b; Kaster and Sobol, 2020; Liu et al., 2021). For example, by using the mainstream MDA amplification method (Spits et al., 2006), researchers have successfully achieved the amplification with 10 fg of *Corynebacterium glutamicum* DNA with a digital microfluidic device, within only 2 h to reach the amount required for successful sequencing (Liu et al., 2021). To be mentioned, with the help of transposase such as Tn5 (Adey, 2021), the DNA fragmentation, end-repair, and adapter ligation steps in traditional workflow of sequencing library preparation, which may cause losses and bias, can be combined to one step called tagmentation, as transposase could added the required adapters while fragmenting the long genomic DNA at the same time. For example, Chen et al. (2017a) reported an isothermal linear amplification method for single-cell DNA amplification called LIANTI, which could achieve both high coverage and uniformity, as well as low allelic dropout rate and false positive rate (Chen et al., 2017a; Adey, 2021). Although methods such as MALBAC and LIANTI have not been successfully applied in microbes, their performance in mammalian cells have proved the potential of better amplification than current methods used in microbes (Zong et al., 2012; Chen et al., 2017a). In summary, amplification of DNA from one microbe is no longer the bottleneck in microbial single-cell analysis, even could be further improved with future applications of several new methods such as MALBAC and LIANTI (Zong et al., 2012; Chen et al., 2017a).

As we previously described (Chen et al., 2017b), single-cell RNA analysis in microbes is more challenging for several reasons, including the low RNA content per cell, the instability of RNA molecules, and most importantly, not all the methods commonly used in mammalian cells can be directly applied on microbes due to the structure differences between eukaryotic and prokaryotic cells. For example, although the new single-cell RNA sequencing platform provided by 10x Genomics has been widely used in mammalians and plants, the successful application of this technique on eukaryotic microbes was very rare (de Bekker et al., 2011; Ma et al., 2021), nor to mention the reports of single-cell RNA profiling in prokaryotes (Kang et al., 2011; Wang et al., 2015; Kuchina et al., 2021). Recently, Ma et al. (2021) have successful applied single-cell RNA sequencing in the photosynthetic unicellular alga *Chlamydomonas reinhardtii*, providing the feasibility of single-cell RNA sequencing in some eukaryotic microbes (Ma et al., 2021). For prokaryotic cell, a method called microSPLiT has been successfully applied to >25,000 *Bacillus subtilis* cells. This method used *E. coli* Poly(A) Polymerase I to polyadenylate the mRNA, thus providing the possibilities of applying the methods capable of eukaryotes to prokaryotic microbes (Kuchina et al., 2021). In addition, Di et al. (2020) reported a new RNA-seq library preparation strategy called SHERRY, which uses Tn5 to tag the RNA/DNA heteroduplex produced in the cDNA synthesis step, provides higher reproducibility and GC uniformity compared with prevailing RNA-seq methods, and could be adapted to single cells after several optimizations (Di et al., 2020). In conclusion, scientists have achieved important progress in single-cell RNA sequencing in microbes, but still face several obstacles to overcome, especially when handling the complex bacterial communities in the wild.

## Future Perspectives

**FIGURE 1:**
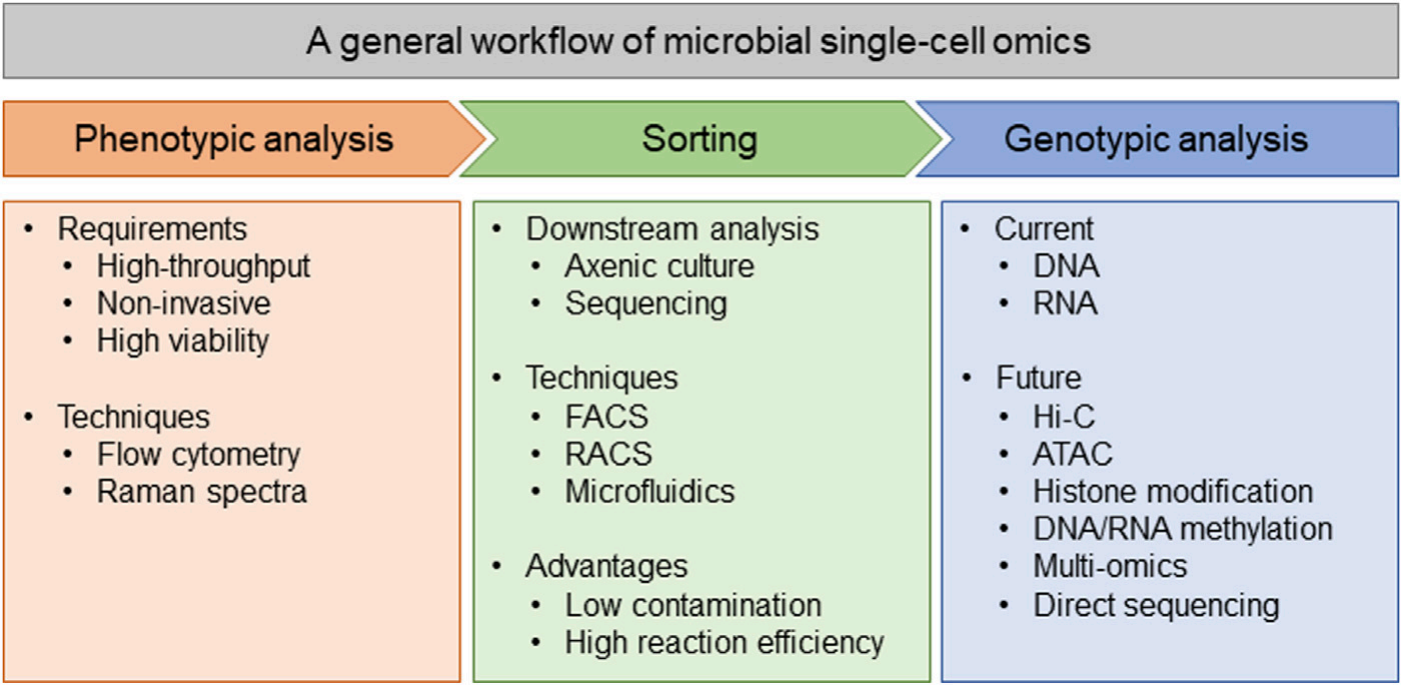
Diagram of the general workflow of microbial single-cell omics.

Generally, a workflow of microbial single-cell analysis includes cell sorting and genotypic analysis, while the sorting step is either phenotype-based selection or random selection (Figure 1). The existence of some commercial sorting methods, such as fluorescence activated cell sorting (FACS) and Raman activated cell sorting (RACS), has made it possible for microbiologists to finish the analysis workflow in a common laboratory. With these sorting methods, scientists can either select microbes with specific phenotype or randomly choose an ideal number of cells, then eject them to agar plates, microplates, or even small oil-water particles for either axenic culture or genotypic analysis. More importantly, if the sorting is phenotype-based, it is possible to unify the phenotype and genotype (genome or transcriptome) in every cell (Xu et al., 2020; Jing et al., 2021). Simultaneously profile phenotype (like metabolites with Ramanome) and genotype (genome or transcriptome) from the same cell could be very helpful in some specific fields, such as finding the related gene clusters for producing specific natural products (Xu et al., 2020; Jing et al., 2021). In addition, the state-of-the-art techniques for single-cell analysis prefer to use microfluidics as the bridge between phenotypic sorting and genotypic analysis (Liu et al., 2021). Using microfluidics, individual cells are sorted into small oil-water particles, together with the regents for cell lysis and further genotypic analysis, which both avoids possible contamination in common tube-based experiments, and improves the reaction efficiency since the concentrations of both nucleic acids and enzymes are raised (Chen et al., 2017b; Liu et al., 2021). In summary, we supposed that an ideal microbial single-cell analysis platform should be high-throughput and composed of both phenotypic sorting and genotypic analysis, with most of the processes being conducted in microfluidics to achieve the best performance (Figure 1).

Besides single-cell DNA and RNA sequencing, recent advances in mammalian single-cell analysis also provided potentials for profiling more genetic information at single-cell level (Zhu et al., 2020). For example, the commercial single-cell ATAC product provided by 10x Genomics makes it possible to obtain the chromatin accessibility of individual cells (Buenrostro et al., 2015; Pott and Lieb, 2015). Methods of single-cell Hi-C could detect the genome-wide chromatin interactions that occur simultaneously in a single cell (Nagano et al., 2013; Nagano et al., 2015; Nagano et al., 2017). The application of the new CUT&Tag technique to single-cells has confirmed the feasibility of generating the histone modifications landscape from single cells, and provides unique insights into epigenomic landscapes (Bartosovic et al., 2021; Wu et al., 2021). In addition, scientists have reported several protocols capable of analysing two or even more “omics” simultaneously in one cell, which provides the possibility of generating complete genetic landscapes from individual cells (Zhu et al., 2020). Moreover, several references have reported successful allele-specific single-cell analysis and lineage tracing in mammalians, which may be capable of studying the sexual reproduction in microbes and reveal some unexpected phenomena and mechanisms in the life cycle of microorganisms (Zhang et al., 2018; Collombet et al., 2020; Weinreb et al., 2020). Furthermore, new techniques developed for bulk cells, such as the CUT&Tag technique for histone modification detection (Kaya-Okur et al., 2019), the EM-seq (Feng et al., 2020; Sun et al., 2021; Vaisvila et al., 2021) and TAPS (Liu et al., 2019) techniques for DNA methylation detection, and DLO-HiC (Lin et al., 2018), BAT Hi-C (Huang et al., 2020) and tagHi-C (Zhang et al., 2020) techniques for improved Hi-C library preparation, may also be capable for single cells, even with several modifications. Finally, current sequencing techniques have made it possible to directly obtain the DNA, RNA, and protein sequences without amplification, making the direct sequencing of individual cells is worth waiting in the coming future (Al Kadi et al., 2021; Brinkerhoff et al., 2021; Mohammadi and Bavi, 2021; Pust et al., 2021; Pyatnitskiy et al., 2021).

In conclusion, we believe that even with many challenges ahead, single-cell analysis in microbes is in its best time, and will receive a tremendous boost based on the progress of several related fields, such as new sequencing platforms, new lab-on-chip devices, and single-cell techniques for mammalians. With the help of single-cell analysis, microbiologists could reveal the mechanisms of how individual microbes perceive, respond and adapt to the complex and changing environment, and finally determine the fate of the whole population.
